# HIV Nef-mediated WAVE2-ARP2/3 inhibition underlies CD4^+^ T-cell lamellipodial abnormalities and immune dysfunction

**DOI:** 10.1128/mbio.03917-25

**Published:** 2026-03-30

**Authors:** Daniel C. Dunn, Jacqueline M. Crater, Perla M. Del Río Estrada, Jeffrey M. Malgady, Griffin Haas, William Klain, Maria Fernanda Torres-Ruiz, Yara Andrea Luna-Villalobos, Mauricio González-Navarro, Chris Kang, Colin Kovacs, Santiago Ávila-Ríos, Benhur Lee, Paul Jolicoeur, Douglas F. Nixon, Robert L. Furler O’Brien

**Affiliations:** 1 Feinstein Institutes for Medical Research, Northwell Health 88982 , Manhasset, New York, USA; 2 Division of Infectious Diseases, Department of Medicine, Weill Cornell Medicine 12295, New York, New York, USA; 3 Centro de Investigación en Enfermedades Infecciosas, Instituto Nacional de Enfermedades Respiratorias Ismael Cosío Villegas 42635, Mexico City, Mexico; 4 Department of Pathology and Laboratory Medicine, Emory University School of Medicine 12239, Atlanta, Georgia, USA; 5 Department of Microbiology, Icahn School of Medicine at Mount Sinai 5925, New York, New York, USA; 6 Abiosciences 699402 , South San Francisco, California, USA; 7 Maple Leaf Medical Clinic, Toronto, Ontario, Canada; 8 Clinical Research Institute of Montreal 5598, Montreal, Quebec, Canada; 9 Department of Microbiology/Immunology, University of Montreal 5622, Montreal, Quebec, Canada; 10 Division of Experimental Medicine, McGill University 5620, Montreal, Quebec, Canada; 11 Gladstone Institute of Virology, Gladstone Institutes 40292, San Francisco, California, USA; Tsinghua University, Beijing, China

**Keywords:** HIV, CD4^+ ^T cell, actin, cytoskeleton, Nef, cell morphology, time-lapse imaging, ARP2/3, WAVE2, Wiskott-Aldrich syndrome, primary immunodeficiencies, P-REX1, mTOR

## Abstract

CD4^+^ T cells are central regulators of adaptive immune responses, and their depletion following HIV infection leads to AIDS. HIV Nef and Gag weaken CD4^+^ T cells by disrupting the actin cytoskeleton, which leads to impaired cell migration and immune synapse formation. Several mechanisms of cortical actin disruption have been proposed; however, a unifying and detailed mechanism has remained elusive. This study investigates how HIV alters the actin cytoskeleton of primary CD4^+^ T cells, aiming to reconcile disparate mechanisms reported in the literature. Using a multi-modal approach combining ultrastructural microscopy, time-lapse imaging, small-molecule inhibitors, and proteomics, we identified potential actin regulators affected by HIV. Two distinct lamellipodial abnormalities were observed following infection, both of which are indicative of ARP2/3 inhibition. These morphologies were highly dependent on Nef *in vitro* and *in vivo*. Direct chemical inhibition of ARP2/3 recapitulated the lamellipodial defects in Nef-expressing cells. Proteomics of primary cells indicates ARP2/3 inhibition at the lamellipodia of infected T cells may occur through Nef-mediated inhibition of the WAVE2 complex, specifically via inhibitory phosphorylation of WASF2. Together with previous reports of WAVE2 regulation in HIV infection, these results support HIV Nef as a major disruptor of cortical actin through WAVE2-ARP2/3 inhibition at the lamellipodia, providing a novel mechanism of HIV-mediated CD4+ T-cell dysfunction and depletion.

**IMPORTANCE:**

CD4^+^ T cells migrate throughout the body and form immune synapses to carry out their functions. Both of these actions require dynamic actin structures, which are disrupted by HIV proteins. Our study suggests that a key HIV protein, Nef, might disrupt a vital internal cellular machinery that helps immune cells move and function properly. Our microscopic and proteomics studies suggest a new model in which Nef inhibits a large protein complex at the front of migrating T cells. Restoring this cytoskeletal dysfunction may be key to restoring CD4^+^ T-cell survival and function, which may improve adaptive immune responses during HIV infection.

## INTRODUCTION

CD4^+^ T cells orchestrate adaptive immune responses to thwart pathogens while dampening hyperactive inflammation seen in autoimmunity (1). Fine-tuning of the immune system is critically dependent on CD4^+^ T cells. When these cells are depleted, profound immunodeficiency ensues. This is observed when T cells are depleted in several primary immunodeficiencies (PID), also known as inborn errors of immunity, which can result from congenital mutations in several actin-related genes (1–4). Similarly, acquired immunodeficiency syndrome (AIDS) occurs following CD4^+^ T cell loss caused by human immunodeficiency virus (HIV) infection. In both AIDS and many PID, depletion of CD4^+^ T cells occurs as a consequence of functional impairment of T-cell migration, immune synapse formation, and/or cell proliferation. One common mechanism underlying cellular dysfunction in several PID and pathogenic infections is disruption of the actin cytoskeleton (2–4).

The actin cytoskeleton is a dynamic structure within eukaryotic cells that, together with myosin and actin-binding proteins, generates the necessary force required for cell migration, attachment, and cell division. The dynamic nature of actin arises from groups of nucleation-promoting factors (NPFs) that build linear or branched actin structures, combined with groups of proteins that disassemble those filaments. Branched actin, formed by a multimolecular complex called ARP2/3, can generate forces on overlying lipid membranes and surrounding structures. ARP2/3 and the force generated by branched actin are critical for lamellipodium formation, clathrin-mediated endocytosis, vesicular trafficking, cellular adhesions, and even DNA repair (3, 5–20). Inhibiting ARP2/3 or its upstream regulators leads to dysfunctional T-cell chemotaxis and immune synapse formation, and congenital mutations in these genes gives rise to several PID (2–4, 21–27).

The ARP2/3 complex is activated by members of the Wiskott-Aldrich syndrome (WAS) family of NPFs, including the distinct WAS and Neural-Wiskott-Aldrich syndrome proteins (WASP and N-WASP), the multimolecular WAVE (WAVE1, WAVE2, and WAVE3) and WASH complexes, along with the WHAMM and JMY proteins. These NPFs translate signals from upstream Rho GTPases (RAC and CDC42) to the ARP2/3 complex. Many intracellular pathogens, including retroviruses, hijack these NPFs or ARP2/3 to create branched actin, which provides the forces needed for cell entry, motility, and propagation (28–31).

HIV significantly disrupts the actin cytoskeleton (32–34) by temporally regulating ARP2/3 (35). At the beginning of the replication cycle, HIV Env induces WAVE2 phosphorylation and subsequent activation of ARP2/3 during direct infection (36). However, lateral transfer of virions from DCs to CD4^+^ T cells was shown to be dependent on WASP (32, 37, 38). Following viral entry, nuclear localization of the viral pre-integration complex may also be dependent on ARP2/3 (36); however, post-integration, later stages of the viral replication cycle require ARP2/3 to be inhibited (39–42). Following infection, cells have reduced F-actin, lose their polarity, reduce their velocity and directionality, fail to traverse endothelial barriers efficiently, and have difficulty migrating in more complex dense environments like peripheral tissues (39, 43–45).

Although multiple HIV proteins are linked to ARP2/3 dysregulation (35), the myristoylated viral proteins Nef (39, 43–49) and Gag (40–42, 50) have repeatedly been shown to alter cortical actin *in vitro* and *in vivo*. Nef has been reported to dysregulate actin through its actions on a Nef-associated kinase complex, PAK2, and cofilin (39, 45, 51, 52). HIV Nef has also been linked to ARP2/3 dysregulation through N-WASP inhibition (43).

Although there is a general agreement that Nef and Gag alter actin dynamics, there are discrepancies in how Nef and Gag may alter ARP2/3 activity. An earlier study indicated HIV Gag required Rac1-WAVE2-ARP2/3 activation via IRSp53 for budding (50). Proteomic identification of WAVE2 (NCKAP1L and CYFIP1) and ARP2/3 (ARPC1B, ARPC4, and ACTR3) components in HIV virions provides additional evidence of potential Gag interactions with the WAVE2 complex (53). However, subsequent studies reported that HIV Gag inhibits ARP2/3 (41), with more recent studies identifying the ARPIN protein (42) or MICAL1 (40) at the center of Gag-mediated ARP2/3 inhibition. Furthermore, there are discrepancies in how HIV Nef alters cell morphology in various studies. Although early reports indicate that HIV infection causes CD4^+^ T cells to lose their polarization on 2D surfaces (45), others showed prolonged polarization with Env-mediated elongated uropods infected cell migration *in vivo* (39, 49). Other reports of infected cells in 3D collagen matrix models showed occasional blebbing. These disparate results are likely due to the cell model used, environmental complexity, image resolution, and time scale in which these experiments were observed.

To decipher the distinct mechanisms often reported from studies using cell lines, we examine ARP2/3 regulation during HIV infection in primary human CD4^+^ T cells and lymph node tissues. Ultrastructural microscopy was combined with time-lapse imaging to confirm the role of Nef in ARP2/3 inhibition at the cellular level. We report two structural abnormalities localized to the lamellipodia in Nef-expressing cells, both of which are newly linked to HIV infection and strongly indicate ARP2/3 inhibition. This was further validated with Nef-expressing T cells from transgenic mice. Spatial transcriptomic profiling of the immune microenvironment at different stages of HIV infection also suggests differential ARP2/3 complex gene expression in viremic lymph nodes. Additionally, to narrow down which reported host factors may mediate Nef and Gag regulation of ARP2/3, bulk and phosphoproteomics studies were done on sorted infected primary cells. Our findings support previous reports that Nef inhibits branched actin within T cells (43); however, not through the neural-specific N-WASP. Our proteomics data indicate that although the neural-specific homolog N-WASP is not expressed in primary human CD4^+^ T cells, the related WASF2 protein (a core component of the WAVE2 complex) is highly expressed and undergoes specific phosphorylation at inhibitory residues with its VCA domain by CK2 in Nef-expressing T cells. Additionally, our bulk proteomics measurements did not detect reported Gag mediators IRSp53 (BAIAP2) (50) or ARPIN (42) in primary T cells, suggesting that if Gag does regulate ARP2/3, it is likely through MICAL1 or other host factors.

## RESULTS

### HIV-infected CD4^+^ T cells exhibit distinct cortical actin disruption and impaired lamellipodial dynamics

**Fig 1:**
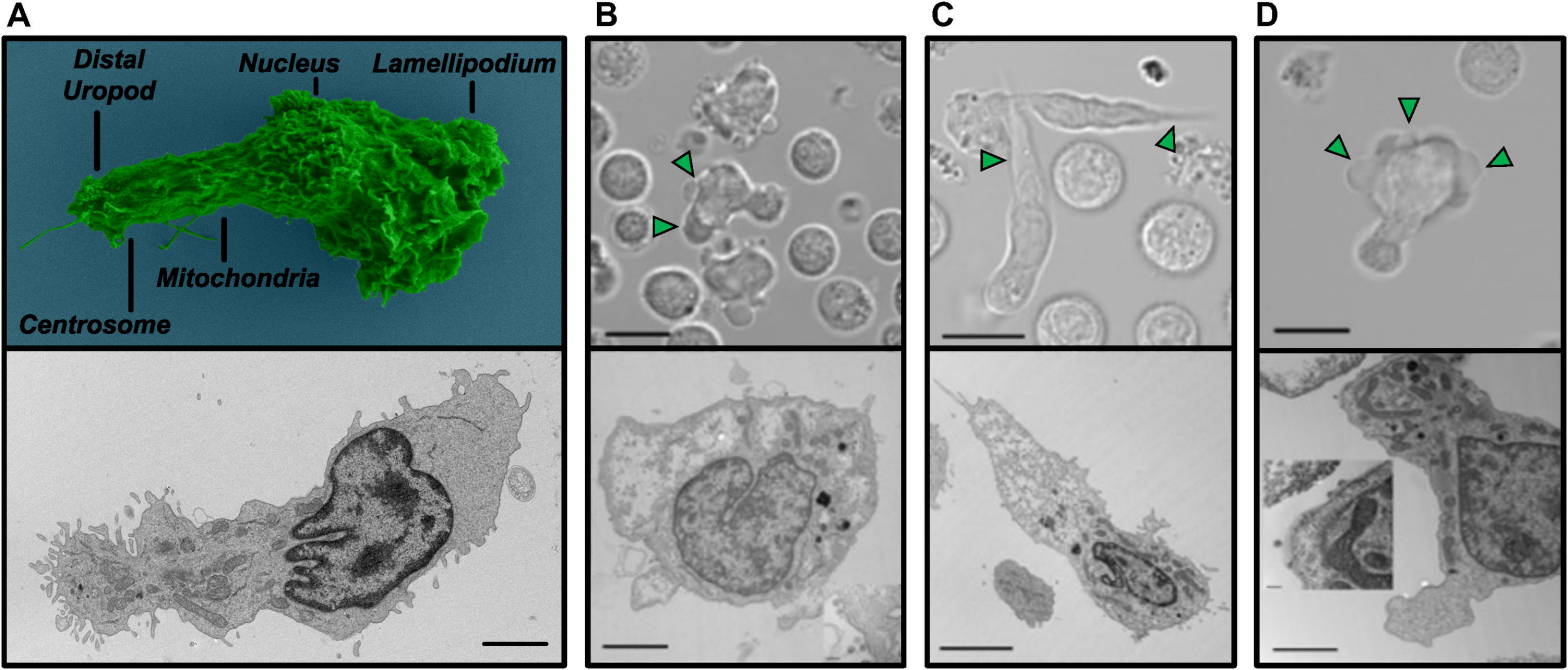
HIV-infected CD4^+^ T cells exhibit distinct lamellipodial abnormalities indicative of cytoskeletal disruption. (**A**) Representative scanning electron microscopy (SEM) and transmission electron microscopy (TEM) of an uninfected, migrating primary human CD4^+^ T cell on a fibronectin-coated surface. The SEM (top) shows a classic polarized morphology with a broad lamellipodium at the leading edge and a trailing uropod. The TEM (bottom, scale = 2 µm) provides a cellular cross-section, highlighting the actin-rich lamellipodium (anterior to the nucleus) and the distribution of organelles away from the leading edge. This morphology is typical of healthy T-cell migration (Video S1). (**B–D**) In contrast to uninfected cells, HIV infection leads to several distinct abnormal cortical actin phenotypes, as observed with time-lapse brightfield imaging and ultrastructural microscopy: (**B**) A rounded cell exhibiting dynamic blebbing (brightfield scale = 10 µm; Video S2). The corresponding TEM (scale = 2 µm) reveals the internal cellular structure during this blebbing, which can occur prior to apoptosis or, in other contexts, virion budding. (**C**) A cell displaying a “Rhino” phenotype (brightfield scale = 10 µm; Video S4). The TEM (scale = 5 µm) shows the abnormal, organelle-void extension, confirming it as a lamellipodial structure distinct from a retracted uropod. (**D**) A cell exhibiting polarized lamellipodial blebbing (brightfield scale = 10 µm; Video S5). The TEM (scale = 2 µm) illustrates the blebbing at the leading edge, with virion production observed at the distal uropod (inset scale = 200 nm), indicating productive infection. These lamellipodial defects suggest impaired ARP2/3 complex activity.

Uninfected migratory CD4^+^ T cells have a polarized morphology with a leading edge or lamellipodium containing dynamic branched and linear F-actin structures that bridge intracellular components to surface adhesion molecules in order to generate migratory forces (Fig. 1A; Video S1) (1, 54). To further characterize cytoskeletal changes within HIV-infected CD4^+^ T cells, ultrastructural microscopy was combined with time-lapse live imaging. Negatively selected CD4^+^ T cells from 15 healthy donors were activated and expanded prior to infection. Following HIV infection, a spectrum of morphological abnormalities emerged in these CD4^+^ T cells. These changes manifested as six distinct structural phenotypes, consistently observed across diverse patient-derived and laboratory-adapted HIV isolates.

Along with normally polarized migratory T cells, there are abundant rounded non-migratory cells in the absence of chemokine addition. These non-polarized cells can easily be seen in both infected and mock conditions and are indicative of the non-polarized infected cells previously reported (45). Two additional structural abnormalities we observed in HIV-infected cells, rounded cell blebbing (Fig. 1B; Video S2) and multinuclear syncytia (Video S2), have previously been reported by multiple groups (33, 55). These first four phenotypes are regularly observed; however, the final two have not been clearly associated with HIV infection.

The last two phenotypes we observed in the infected cultures exhibited abnormal lamellipodia. The first lamellipodial defect observed was an elongated and pointed lamella/lamellipodia, sometimes, but not exclusively, accompanied by a retracted uropod, a morphology previously described as a “Rhino” phenotype (56) (Fig. 1C; Video S3). To distinguish the lamellipodial structure from the uropod, vital dyes recognizing the nucleus and mitochondria were introduced to the culture. The elongated structure was anterior to the nucleus and void of membrane-bound organelles typically localized to the uropod, indicating the abnormal extension is indeed the leading edge of the cell. The “Rhino” phenotype is an abnormal pointed elongation of the lamella/lamellipodium and should not be confused with uropod retraction defects previously shown to be HIV Env dependent in lymphoid tissues (49). The second lamellipodial defect was nonapoptotic polarized blebbing. These cells have typical extended uropods and produce virions at the distal uropod (Fig. 1D; Video S4). Although this phenotype was observed in two-dimensional cultures during this study, similar blebbing was reported in HIV-infected T cells migrating in three-dimensional collagen matrices (39).

### HIV-associated cytopathologies and lymph node gene expression profiles indicate ARP2/3 inhibition

To identify potential mechanisms underlying the two distinct lamellipodial defects observed in HIV-infected cultures, previous reports detailing cytoskeletal deformities outside the context of viral infection were investigated. Elongated phenotypes were reported in migrating leukocytes following depletion of the actin regulators DOCK8, CDC42, and PAK1/2 (57); however, these leukocytes have drastically elongated nuclei, which we did not observe in HIV-infected CD4^+^ T cells. Inhibiting many of these actin regulatory proteins, including CDC42, Rac1/1b/2/3, and PAK2, induced varied phenotypes that do not resemble HIV-infected cells (Video S5). On the contrary, there are numerous reports outlined in Table 1 that mirror the two lamellipodial defects we observed in HIV-infected cultures. Polarized lamellipodial blebbing (19, 39, 58–73) and the “Rhino” phenotype (20, 41, 56, 63, 66, 74) have been described in multiple cell models from different species, all with a unifying mechanism surrounding ARP2/3 complex inhibition. Supplemental videos can be found in many of these published reports that align with the phenotypes we see in HIV-infected cultures. Of particular interest are several mutations in T cells and other leukocytes that affect ARP2/3 or the WAVE2 complex, including the *Actr3* knockdown phenotype in murine CD8^+^ T cells (19) and the phenotype of primary CD4^+^ T cells from pediatric patients with a deficiency in *HEM1*, a component of the WAVE2 complex. The phenotypes in reports listed in Table 1 mirror those seen in HIV-infected cells. The common mechanism of ARP2/3 in all of these reports strongly supports the hypothesis that the lamellipodial defects observed in HIV-infected cultures likely arise from an inhibition of ARP2/3 or an upstream regulator.

**TABLE 1:** Summary of reported lamellipodial defects mirroring HIV infection

Affected protein complex	Mutation, knockdown, or chemical Inhibition	Specific target	Cell type	Organism	Citation
Polarized lamellipodial blebbing					
Rho GTPases	Lamellipodium forming (LC) vs. spontaneously blebbing (BC)	RAC/CDC42/RHO signaling	Walker 256 carcinoma-sarcoma cell line	*Rattus norvegicus*	(58)
ARP2/3	Small-molecule inhibitor CK-666	ARP2/3	HT1080 fibrosarcoma cell line	*Homo sapiens*	(59)
ARP2/3	Small-molecule inhibitor CK-666	ARP2/3	Walker 256 carcinoma-sarcoma cell line	*Rattus norvegicus*	(60)
ARP2/3	siRNA knockdown	*ARPC2*	HeLa cell line	*Homo sapiens*	(61)
ARP2/3	Conditional gene knockout	*Arpc2* ^−/−^	Primary bone marrow-derived macrophages	*Mus musculus*	(62)
ARP2/3	shRNA knockdown	*Actr3*	CD8^+^ T cells	*Mus musculus*	(19)
ARP2/3	Conditional gene knockout	*Arpc4* ^−/−^	Mast cells	*Mus musculus*	(63)
ARP2/3	Knockout	*Arpc4* ^−/−^	Keratinocytes	*Mus musculus*	(64)
ARP2/3	Small-molecule inhibitor CK-666	ARP2/3	Neutrophils	*Mus musculus*	(65)
ARP2/3 or WAVE2	CRISPR-Cas9 knockout	*ARP2*/*ACTR2*^−/−^ or *HEM1*/*NCKAPL1*^−/−^	HL-60 neutrophil cell line	*Homo sapiens*	(66)
WAVE2	siRNA knockdown	*BRK1*	HeLa cell line	*Homo sapiens*	(61)
WAVE2	siRNA knockdown	*WASF2*	HeLa cell line	*Homo sapiens*	(61)
WAVE2	siRNA knockdown	*WASF2*	Primary dermal microvascular endothelial cells	*Homo sapiens*	(67)
WAVE2	siRNA knockdown	*NCKAP1* (*NAP1*)	HeLa cell line	*Homo sapiens*	(61)
WAVE2	IEI/PID patient	*NCKAP1L* (*HEM1*)^−/−^	Primary CD4^+^ T cells	*Homo sapiens*	(68)
WAVE2	siRNA knockdown	*Sra-1* or *Nap1*	B16-F1 melanoma cell line	*Mus musculus*	(69)
WAVE2	Knockout	*scar* ^−^	Dictyostelium Ax3 cells	*Dictyostelium discoideum*	(70)
WAVE, WASP, and formin	Triple knockout	*scar*^−^ *wasA*^−^ *ddia2*^−^	Dictyostelium Ax3 cells	*Dictyostelium discoideum*	(71)
Formin	Overexpression-transfection	FMNL1γ and FMNL1ΔDAD variants	HEK293 cell line	*Homo sapiens*	(72)
Formin	Adenoviral transduction	FMNL1γ and FMNL1ΔDAD variants	K562 cell line	*Homo sapiens*	(72)
NEDD9	Knockout	CasL^−/−^	Primary CD4^+^ T cells	*Mus musculus*	(73)
			Primary CD4^+^ T cells—HIV infected	*Homo sapiens*	(39)
Rhino phenotype					
ARP2/3	Conditional gene knockout	*Arpc4* ^−/−^	Mast cells	*Mus musculus*	(63)
ARP2/3 or WAVE2	CRISPR-Cas9 knockout	*ARP2*/*ACTR2*^−/−^ or *HEM1*/*NCKAPL1*^−/−^	HL-60 neutrophil cell line	*Homo sapiens*	(66)
WAVE2	Conditional deletion gene knockout	*Hem1* ^−/−^	Primary dendritic cells	*Mus musculus*	(20)
WAVE2	CRISPR-Cas9 knockout	*WASF2* ^−/−^	U937 monocytic cell line	*Homo sapiens*	(41)
WASP	shRNA knockdown	*WAS*	HL-60 neutrophil cell line	*Homo sapiens*	(56)
N-WASP	siRNA knockdown	*WASL*	A375M2 melanoma cell line	*Homo sapiens*	(74)

**Fig 2:**
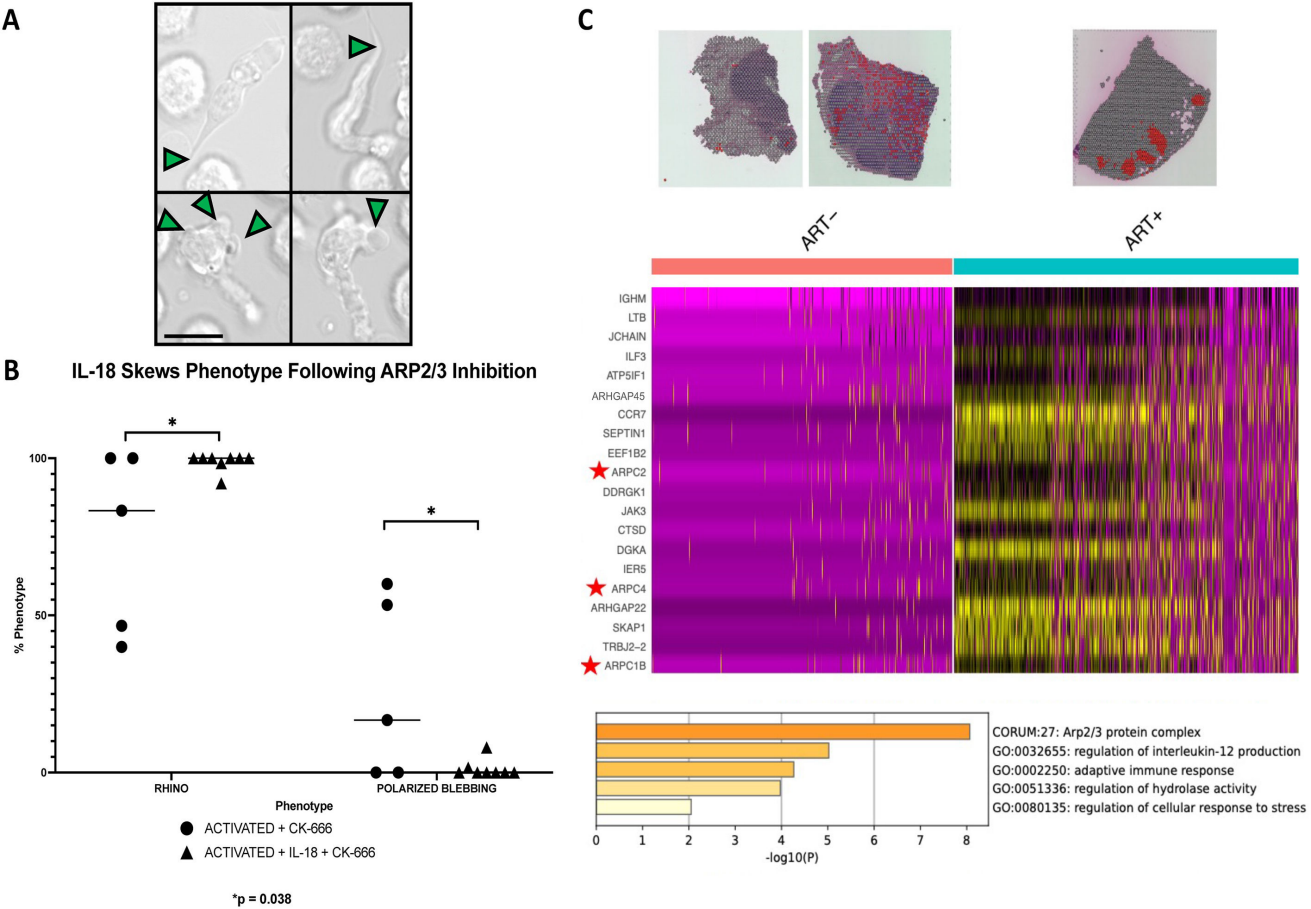
HIV-infected CD4^+^ T cells exhibit signatures of ARP2/3 inhibition. (**A**) Time-lapse brightfield images illustrating the morphological consequences of chemical ARP2/3 inhibition in uninfected primary human CD4^+^ T cells. Treatment with the small-molecule inhibitor CK-666 (200 µM) leads to the emergence of both the polarized lamellipodial blebbing and the "Rhino" phenotypes, recapitulating the abnormalities observed in HIV-infected cells (scale = 10 µm; Video S5). This direct chemical inhibition supports the hypothesis that HIV-induced lamellipodial defects arise from ARP2/3 dysfunction. (**B**) The two distinct lamellipodial defects ("Rhino" and polarized blebbing) observed in ARP2/3-inhibited and HIV-infected T cells can be modulated by differentiation status. Pre-differentiating T cells with IL-18 prior to infection enhances the "Rhino" phenotype, suggesting a maturational link between these two populations and their susceptibility to specific morphological defects. This finding aligns with previous WAVE2 complex knockout studies in dendritic cells and other leukocytes. (**C**) Spatial transcriptomics analysis of lymph node tissues from people living with HIV (PLWH) reveals transcriptional evidence of ARP2/3 complex downregulation during viremia. Comparison between virally suppressed PLWH (on ART) and viremic PLWH (ART-naïve) showed decreased expression of multiple actin cytoskeletal genes, specifically ARPC1B, ARPC2, and ARPC4 (three members of the ARP2/3 complex), within CD4+ T cell clusters (Red spots) in viremic lymph nodes (top 20 downregulated genes shown, full list in Table S1B). Metascape was used for gene ontology analysis of the top DEGs, which showed the ARP2/3 complex as the most significantly downregulated ontology in the viremic tissues compared to ART-treated tissues. This gene expression signature further supports broad ARP2/3 dysfunction in lymphoid tissues during active HIV infection.

Previous reports indicate that membrane blebbing in other cell types can be induced by disrupting the branched cortical actin structures in human cells through chemical inhibition of the ARP2/3 complex with the small-molecule inhibitor CK-666 (59, 60, 75). Since the phenotypes of ARP2/3 inhibited cells from other studies strongly mirror the aberrant morphologies we observed in HIV-infected cells, we characterized the morphology of primary human CD4^+^ T cells following chemical inhibition of the ARP2/3 complex with CK-666. Following the addition of the small-molecule inhibitor of ARP2/3, CK-666, both polarized lamellipodial blebbing and the “Rhino” phenotype were observed in uninfected CD4^+^ T cells (Fig. 2A; Video S5).

The presence of two aberrant morphologies we see in primary CD4^+^ T cells following ARP2/3 inhibition is consistent with the WAVE2 complex knockout studies previously done in dendritic cells (20). That study concluded that these two morphologies were dependent on dendritic cell maturation status and tissue distribution. Immature dendritic cells without the WAVE2 complex were found in peripheral tissues and exhibited the “Rhino” phenotype, whereas TLR-stimulated dendritic cells, which differentiate as they migrate into lymphatic tissues, exhibited a second morphology when they lacked the WAVE2 complex. This suggests that the two observed lamellipodial defects may be dependent on the maturation status of the CD4^+^ T cell. Our findings indicate that IL-18, whose receptor is related to TLRs, enhances the “Rhino” phenotype in ARP2/3-inhibited T cells (Fig. 2B), suggesting a maturational link between these two populations.

Additional evidence of ARP2/3 repression was found in lymphatic tissues from people living with HIV (PLWH). Differentially expressed genes were spatially quantified in CD4^+^ T cells found within intact lymph nodes. Four serial sections of lymph node tissues from two viremic PLWH were compared to four serial sections of a lymph node from a PLWH virally suppressed with ART (Fig. 2C; Table S1). The 12 samples were merged into a single Seurat object, normalized, and variable features were identified. The data set was then scaled and subjected to principal component analysis to reduce dimensionality and facilitate downstream analyses. Spots containing CD4^+^ T cells were analyzed for the top 20 DEGs between the ART-naïve and virally suppressed tissue sections. Metascape was used for gene ontology analysis of the top DEGs (76). The ART-naïve tissue sections from viremic PLWH had decreased expression of multiple actin cytoskeletal genes in the top 10 downregulated genes, three of which were members of the ARP2/3 complex: *ARPC1B, ARPC2,* and *ARPC4*.

The common lamellipodial defects observed in ARP2/3-inhibited and HIV-infected T cells, combined with the gene expression signatures in lymphoid tissues, consistently point toward a broad ARP2/3 dysfunction impacting the lamellipodia during HIV infection.

### HIV Nef is a key viral determinant responsible for ARP2/3 inhibition

**Fig 3:**
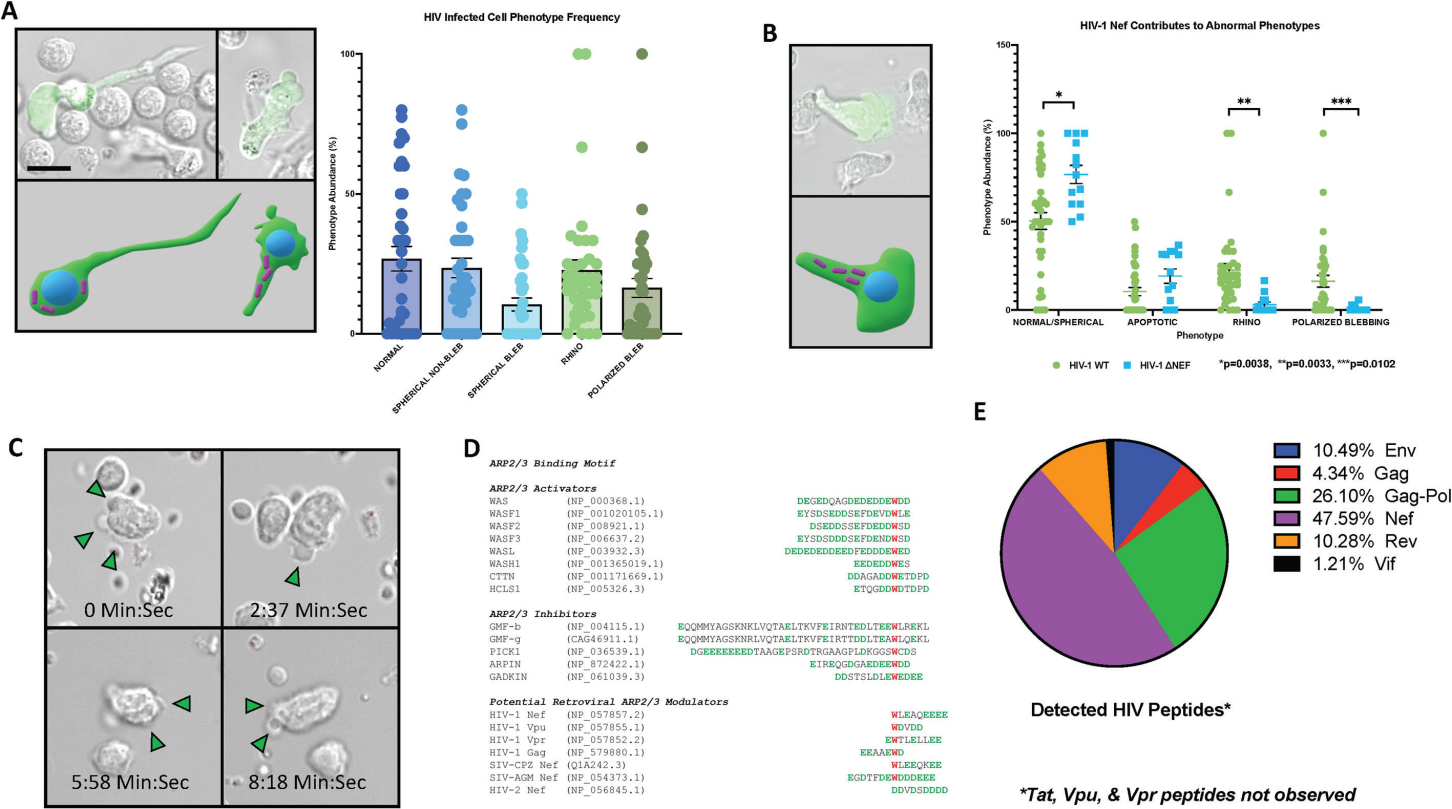
HIV Nef is a key determinant for ARP2/3 inhibition and associated morphological changes in CD4^+^ T cells. (**A**) Time-lapse brightfield microscopy demonstrates that the "Rhino" and polarized lamellipodial blebbing phenotypes are specific to productively infected cells (EGFP+, infected with HIV WT IRES-EGFP reporter virus). Neighboring uninfected cells (EGFP−) maintain normal migratory morphologies (Video S6), indicating that ongoing viral replication is essential for these cortical actin disruptions. Cartoons depict the observed morphologies with the anatomical orientation of the nucleus (blue) and mitochondria (fuchsia rods) for clarity. (**B**) The absence of the Nef protein reduces lamellipodial disruption. Primary CD4^+^ T cells infected with a ΔNef IRES-EGFP reporter virus exhibit largely restored polarization and lamellipodial structure (scale = 10 µm; Video S8). While the frequency of abnormal morphologies is significantly reduced, some residual polarized blebbing still occurs, suggesting potential contributions from other viral factors to actin instability. (**C**) Expression of HIV Nef alone is sufficient to induce lamellipodial defects. CD4^+^ T cells isolated from the spleens of CD4C/HIV Nef transgenic mice, which constitutively express Nef in CD4^+^ T cells, display polarized blebbing similar to human T cells infected with wild-type HIV (Video S7). This confirms Nef’s direct role in causing these cortical actin abnormalities. Non-transgenic control cells exhibited standard morphologies of non-infected CD4^+^ T cells (data not shown). (**D**) Screening of HIV proteins for an ARP2/3 regulatory motif (tryptophan residue surrounded by a polyacidic region) identified potential motifs in Nef, Gag, Vpr, and Vpu. (**E**) However, mass spectrometry of sorted HIV-infected human CD4^+^ T cells only detected significant levels of Nef and Gag. Given their known roles in actin disruption, Nef and Gag are strong candidates for mediating ARP2/3 dysfunction in infected cells.

HIV infection consistently induced these cytopathologies in primary T cells despite infection with various R5 and R5/X4 tropic viruses, including ADA, HIV 92/BR/014, and HIV 92/BR/004. To confirm that these abnormal morphologies were occurring within HIV-infected cells rather than bystander cells, cultures were infected with a fully intact R5 tropic virus containing both the *nef* gene and an IRES-EGFP reporter (39). As shown in Fig. 3A; Video S6**,** these cortical actin abnormalities were unique to the infected (EGFP^+^) cells. Neighboring uninfected cells migrate normally with unaltered morphologies, suggesting ongoing viral replication is key to the observed cortical actin disruptions.

To assess the role of Nef in the aberrant morphologies, we characterized infected cell morphology following infection with wild-type (WT ) (Fig. 3A; Video S6) and Nef-deleted (ΔNef) IRES-EGFP reporter viruses (Fig. 3B; Video S7). Although infection with ΔNef almost completely restored wild-type polarization and lamellipodial structure, nonapoptotic blebbing still occurred, albeit at a decreased frequency. To further assess the effect of HIV Nef on primary T-cell movement independent of the remaining HIV proteins, CD4^+^ T cells were isolated from the spleens of CD4C/HIV Nef transgenic mice, also known as MutG (77). These mice, bred on the C3H background, express only Nef constitutively in CD4^+^ T cells and myeloid cells and display AIDS-like pathologies. Purified Nef-expressing CD4^+^ T cells from these mice exhibited polarized blebbing while migrating, similar to human T cells infected with full-length virus (Fig. 3C; Video S7)**,** indicating Nef is sufficient to cause these cortical actin abnormalities *in vivo*.

This suggests that HIV Nef is a major inhibitor of ARP2/3 at the lamellipodia; however, there are possibly additional viral mechanisms outside of Nef that induce actin instability. ARP2/3 regulatory proteins are known to have a common motif consisting of a tryptophan residue surrounded by a polyacidic region known to interact with ARP2/3 (Fig. 3D). Screening of HIV proteins for this motif indicated some isolates of HIV Nef, Vpu, Vpr, and Gag all contain potential ARP2/3 regulator motifs; however, only Nef and Gag were readily detected in downstream proteomics analysis of sorted infected cells, making these membrane-localized viral proteins the key candidates as ARP2/3 regulators (Fig. 3E). Our data, along with the previous reports of Gag, Nef, and Env regulating ARP2/3 activity during the replication cycle (31, 36, 40–42, 50), provide further evidence that HIV targets ARP2/3 at multiple stages in the viral replication cycle.

### HIV Nef stimulates phosphorylation of a lamellipodial complex that centers on WAVE2-ARP2/3 Regulation

Prior reported mechanisms of HIV-mediated ARP2/3 inhibition include Nef regulation of N-WASP (43) and Gag co-opting IRSp53 (BAIAP2) (50), ARPIN (42), or MICAL1 (40). Bulk proteomic analysis of sorted infected cells was done to identify potential ARP2/3 components or regulators that may be involved in HIV-mediated ARP2/3 inhibition. Label-free mass spectrometry was used to quantify proteins that were differentially expressed in sorted HIV WT, HIV ΔNef, and mock-infected primary CD4^+^ T cells. A total of 5,559 proteins were identified, including six HIV proteins and EGFP in ten biological replicates (Table S2B). All ARP2/3 components and upstream NPFs, including WASP and the WAVE2 and WASH complexes, were found in all conditions. However, the previously reported mediators of HIV cortical actin disruption—N-WASP, IRSp53 (BAIAP2), and ARPIN—were not detected in this data set. Only MICAL1 was present in our proteomic screen of primary human CD4^+^ T cells. This discrepancy may be due to different T-cell models or culture conditions used in previous studies. However, the microscopic and proteomics data presented here suggest alternate mechanisms for ARP2/3 inhibition by Nef and potentially Gag in primary CD4^+^ T cells.

The sustained presence of the ARP2/3 complex and its upstream NPFs during infection suggests ARP2/3 may be inhibited by non-degradative means, potentially through post-translational modifications (78–80). To gain further insight into potential mechanisms of ARP2/3 inhibition, primary CD4^+^ T cells were infected with wild-type or ΔNef EGFP reporter HIV, sorted by infection status, and analyzed by phosphosite mass spectrometry (Table S3A through D). There were 192 proteins that had unique phosphosites detected in cells infected with the Nef-expressing virus. As expected with known Nef functions, multiple proteins (e.g., DNM2, SYNJ1, DBNL) involved in clathrin-mediated endocytosis (CME) were detected in this screening, but are adjacent to the focus of the current study. CME utilizes a CDC42-WASP-ARP2/3 pathway (81–85), which suggests Nef may be rerouting ARP2/3 from a membrane protrusive Rac-WAVE2 function to a CDC42-WASP endocytic function.

Although ARP2/3 complex proteins did not have preferential phosphorylation changes within Nef-expressing cells, multiple lamellipodial actin drivers are differentially phosphorylated in Nef-expressing cells. The WASF2 protein (the component of the WAVE2 complex that activates ARP2/3) had several unique phosphosites in the VCA domain exclusively in Nef-expressing cells. These sites are targeted by CK2 and were previously reported to cause inhibition of the WAVE2 complex (70, 86–88).

There are several additional lines of evidence that point to the WAVE2 complex in the underlying mechanism of the lamellipodial defects seen in Nef-expressing T cells. First, several WAVE2 complex knockdown and knockout experiments on leukocytes phenocopy both of the lamellipodial defects observed in HIV-infected cells (20, 66, 70, 71). Second, multiple murine models with conditional knockouts of the WAVE2 complex show immunological characteristics similar to AIDS, including inverted CD4/CD8 ratios, skewing of T cells toward exhausted memory phenotypes, and enhanced autoimmunities (20, 89). This is further exemplified in humans through recently identified genetic mutations in the *NCKAP1L (hem1*) gene of the WAVE2 complex. These patients have a primary immunodeficiency and exhibit recurrent infections and increased autoimmunity (24, 25). Primary migrating CD4^+^ T cells from these patients also exhibited polarized lamellipodial blebbing identical to what we observe in HIV-infected T cells (24).

In the context of HIV infection, the WAVE2 complex is initially activated by Env during the entry/fusion stage (31, 36); however, there is evidence that WAVE2 is detrimental to later stages of the viral replication cycle. One proteomics study investigated host proteins that were specifically downregulated from CD4^+^ T-cell plasma membranes in the presence of HIV Nef (90). Three of the key WAVE2 complex members—WASF2, CYFIP2, and NCKAP1L—were among the 135 identified proteins that were downregulated from the plasma membrane when Nef was transduced into CEM T cells (90). In a separate study, five of the WAVE2 complex members (WASF2, ABI1, NCKAP1, CYFIP1, and BRK1) were identified as drivers of HIV latency in siRNA screens of genes that affected HIV transcription (91). Collectively, these studies reveal that while the WAVE2 complex is critical for initial HIV entry through Env-mediated activation, later in the replication cycle, Nef promotes the specific downregulation of key WAVE2 components from the plasma membrane, linking WAVE2-ARP2/3 manipulation to the establishment of HIV latency.

A binding partner of the WAVE2 complex, mTOR was also identified as an opposing latency repressor in that same screen (92). mTOR is a known driver of HIV replication (93). Multiple reports have provided a cross-regulatory complex between mTOR and WAVE2 through direct binding (68, 89, 94), suggesting a potential link between WAVE2-ARP2/3 regulation and metabolic status of the cell. The RAC-GEF protein P-REX1 also links WAVE2 activation to mTOR. In addition to WASF2, the upstream P-REX1 protein was also uniquely phosphorylated in the Nef-expressing cells. P-REX1 is a unique RAC-GEF that requires two signals to activate RAC (95–100). The first is the binding of PI_(3,4,5)_P_3_, which requires PI3K signaling from neighboring adhesions or growth factor receptors. The second signal P-REX1 requires is the binding to Gβγ proteins downstream from an activator GPCR like CCR5 or CXCR4. In addition to activating RAC at the lamellipodia, P-REX1 also directly binds the mTORC2 complex (101–103). The common binding partners suggest that a larger multimolecular complex (P-REX1, Rac, WAVE2, and mTORC2) acts at PI_(3,4,5)_P_3_ lamellipodial membranes and translates extracellular nutrient sensing (growth factors, chemokines, cytokines, and adhesions) into mTOR activation and WAVE2-ARP2/3-mediated actin branching. This multimolecular lamellipodial complex bridges chemokine and growth factor receptor signaling to adhesions and culminates in WAVE2-ARP2/3 activation. It is possible that these adhesion signaling complexes are disrupted by HIV Nef and the lamellipodia, which dysregulate CD4^+^ T cell migration, metabolism, and function.

In an initial test of this nutrient-sensing module, we used published small-molecule inhibitors of the P-REX1 protein (104, 105) to block this potential complex in uninfected primary human CD4+ T cells and CEM T4 cells. Similar to inhibiting other proteins in this larger complex (ARP2/3, WAVE2, and CASL), inhibition of P-REX1 led to the same lamellipodial defects seen during HIV infection (Video S8).

In addition to inhibitory phosphorylations of WASF2 and P-REX1, several binding partners and upstream actin regulators were also uniquely phosphorylated in the Nef-expressing infected cells. The RAC-GAPs ARHGAP15 and BCR also had multiple unique phosphosites detected. The Ena/VASP family protein EVL, ZYX, and the RAPH1/Lamellipodin homolog APBB1IP were all uniquely phosphorylated in Nef-expressing cells. These three proteins bind to WAVE2 to drive lamellipodial actin protrusion at adhesions (106, 107). Many of these proteins either directly bind each other or work cooperatively in a larger complex at the lamellipodia. The commonality between many of these differentially phosphorylated proteins involves bridging chemokine or nutrient sensing to cellular adhesions (e.g., integrins or TCR).

## DISCUSSION

A key finding in this study is that polarized lamellipodial blebbing and “Rhino” morphological phenotypes are specific to productively infected CD4^+^ T cells. The “Rhino” phenotype and nonapoptotic polarized lamellipodial blebbing, similar to the phenotypes of HIV-infected cells we observed in this study, occur following mutations in actin regulatory proteins that affect ARP2/3 activation (Table 1). These abnormalities are observed regardless of infection with various HIV reporter and primary viral isolates and are predominantly but not exclusively due to Nef. HIV-infected cells strikingly mirror cells with defective ARP2/3-mediated actin branching. Cells with inhibited ARP2/3 activity, whether induced by small-molecule inhibitors, gene knockdowns, or the presence of negative protein regulators, exhibit both “Rhino” and polarized lamellipodia blebbing phenotypes (20, 56, 108). We found that the HIV Nef protein induced phenotypes indicative of WAVE2-ARP2/3 inhibition *in vitro* and also in an *in vivo* mouse model. This is further supported by two key studies showing Nef-mediated downregulation of WAVE2 complex members from the plasma membrane and the role of WAVE2 in HIV latency (90, 91).

Potential mechanisms of HIV-induced ARP2/3 dysregulation are still under investigation. Although WAVE2 and ARP2/3 functionality was previously shown to be required for the early steps of HIV infection (31, 36), others have indicated that ARP2/3 functionality may be altered at the budding stages later in the replication cycle (39, 109, 110). Our results further support a model where both HIV Nef and Gag, which are both membrane-localized N-myristoylated proteins, may work synergistically to regulate ARP2/3 in infected cells.

The inhibition of ARP2/3 provides a mechanistic link between HIV infection and the induction of a senescence-like phenotype (111). Many key signaling pathways are activated in both immunosenescence and HIV-infected cells (92), including NFkB, p38 MAPK, cGAS-STING, and mTOR, all of which can be activated by ARP2/3 inhibition. ARP2/3 inhibition was previously reported to induce activation of both NF-κB and p38 MAPK signaling pathways (111), consistent with their known roles in inflammation and senescence, further supporting the link between Nef-mediated ARP2/3 regulation and HIV-induced senescence. Additionally, increased DNA damage and subsequent activation of the cGAS-STING pathway in HIV-infected cells have been reported (112, 113), possibly stemming from impaired homology-directed repair (HDR) of dsDNA damage due to ARP2/3 dysfunction. This finding aligns with reports demonstrating the importance of ARP2/3 in maintaining genomic stability and preventing cellular senescence (17, 111, 114). Interestingly, the ARP2/3 activator WAVE2 has been shown to inhibit mTORC2 (89), and mutations in WAVE2 complex genes lead to mTOR hyperactivation, suggesting a potential interplay between ARP2/3, mTOR signaling, and senescence. While our findings highlight the possibility of Nef-mediated ARP2/3 inhibition in HIV-induced senescence, the roles of other viral proteins like Env and Gag, which also interact with the actin cytoskeleton, warrant further investigation. Further research is needed to fully decipher the downstream consequences of Nef-mediated ARP2/3 inhibition and its contribution to the immunosenescence observed in individuals with HIV, even during ART-mediated viral suppression.

While our findings suggest a strong link between HIV Nef and WAVE2-ARP2/3 inhibition at the lamellipodia, several limitations should be considered. Although we demonstrated transcriptional downregulation of ARP2/3 in lymphoid tissues during viremic conditions and potential inhibitory phosphorylation of the WAVE2 complex, we lack direct evidence of how Nef induces CK2-mediated phosphorylation of the WAVE2 complex. Furthermore, our study relied primarily on *ex vivo* and *in vitro* models, and further *in vivo* studies are necessary to confirm the physiological relevance of Nef-mediated WAVE2-ARP2/3 inhibition in the context of HIV infection. The sample sizes for some experiments, particularly the human tissue analyses, were limited, potentially affecting the generalizability of our findings.

Viral proteins, including Nef and downstream effectors PAK2 and cofilin, have been implicated in actin disruption with varied effects on leukocyte morphology and migration (51, 52). The role of PAK2, cofilin, or other Nef-associated enzymes in the possible inhibition of WAVE2, ARP2/3, or its upstream regulators needs to be further examined. The PAK2-binding residue of Nef (F191) was reported to be required for Nef-mediated cortical actin disruption; however, this was not tested in our study (39). The WAVE2 component ABI1 has been reported to be directly phosphorylated and inhibited by the Nef-binding partner PAK2 (115); however, we did not detect these PAK2-specific phosphosites in our study. Although our data suggest involvement of CK2 in inhibiting WAVE2-ARP2/3 at the lamellipodia, Nef may still inhibit WAVE2-ARP2/3 activity through PAK2 and cofilin in a yet undefined mechanism. The known role of Nef in CME also suggests that Nef could be rerouting ARP2/3 activity away from WAVE2 at the lamellipodia to WASP at sites of CME.

In summary, this investigation provides additional evidence that HIV Nef exploits the WAVE2-ARP2/3 complex, a cornerstone of dynamic actin remodeling, to induce severe lamellipodial abnormalities and consequent immune dysfunction in CD4^+^ T cells. We demonstrated that Nef-expressing cells exhibit characteristic cytoskeletal defects, which are recapitulated by direct ARP2/3 inhibition and correlate with inhibitory phosphorylation patterns within the WAVE2 complex. Moreover, the observed transcriptional downregulation of ARP2/3 components in viremic lymph nodes reinforces the clinical relevance of this viral strategy. This Nef-mediated subversion of WAVE2-ARP2/3 not only impairs T-cell migratory capacity and immune synapse formation but also establishes a mechanistic link to hallmarks of HIV immunopathogenesis, including chronic inflammation and cellular senescence through intricate crosstalk with pathways like mTOR. Unraveling the specific mechanisms by which Nef mediates CK2-dependent phosphorylation of the WAVE2 complex and further investigating the interplay with other viral proteins like Gag will be the next crucial steps. Nevertheless, targeting this newly identified Nef-WAVE2-ARP2/3 axis potentially offers a new strategy to restore crucial CD4^+^ T-cell functions and improve long-term outcomes for individuals affected by HIV.

## MATERIALS AND METHODS

### CD4^+^ T-cell isolation and activation

Peripheral blood samples from deidentified participants were purchased from the Gulf Coast Blood Center (Houston, TX, USA). Freshly acquired blood was processed by Ficoll-Paque separation to isolate peripheral blood mononuclear cells (PBMC) from healthy donors and HIV progressors. Negative selection of total CD4^+^ T cells was done using StemCell EasySep Human CD4^+^ T Cell Enrichment Kit (Catalog #19052). Cells were then cultured in RPMI, 10% fetal bovine serum (FBS), penicillin, streptomycin, and glutamine at 1–2 million cells/mL. Infections were done on negatively selected total human CD4^+^ T cells that were previously activated with anti-CD3 (UltraLEAF Purified antihuman CD3 Antibody, clone OKT3, Biolegend, Catalog #317325) and anti-CD28 (UltraLEAF Purified antihuman CD28 Antibody, Clone CD28.2, Biolegend, Catalog #302933) antibodies in the presence of rhIL2 and IL15. The medium was changed every 3–4 days for 2 weeks prior to infection. Post-activation and prior to infection, a second round of negative selection was done to achieve 97-99 % CD4^+^ T-cell purity as measured by flow cytometry. The cells were then resuspended at a concentration of 2 × 10^7^ cells/ml in RPMI supplemented with 10% FBS, penicillin, streptomycin, glutamine, IL2, and IL15 prior to infection.

### HIV infections

Primary human CD4+ T cells, previously isolated and activated as described above, were infected with HIV-1 isolates. For each infection condition, 500 µL of cells (at a concentration of 2 × 10^7^ cells/mL) were combined with 500 µL of virus supernatant (or mock supernatant for control conditions) in a well of a six-well plate. Infections were performed at a multiplicity of infection (MOI) of 1, as determined by the infectious titer (TCID50) or p24 content of the viral stocks. To enhance infection efficiency, the cell-virus mixture was spinoculated by centrifugation at 2,900 rpm (1,965 × *g*) for 2 h at 37°C. Following spinoculation, the infected cells were gently resuspended and combined with an equal number of non-spinoculated (but similarly pre-activated) CD4^+^ T cells from the same donor. The combined cells were then cultured at a density of 2 × 10^6^ cells/mL in complete RPMI medium (supplemented with IL-2 and IL-15 as described previously) at 37°C. Cultures were maintained for up to 3 weeks, with media changes and microscopic analysis performed every 2–3 days.

For experiments requiring increased infected cell populations (e.g., for subsequent mass spectrometry analyses), cells were optionally concentrated on day 3 post-infection. This involved transferring the cultures into 96-well U-bottom plates and culturing them at a density of 2 × 10^6^ cells/mL in 200 µL per well for an additional 3–4 days.

The percentage of infected cells in the cultures was routinely monitored. For wild-type and primary isolates, infection rates were assessed by flow cytometry, measuring intracellular HIV Gag p24. When using reporter viruses, infection was monitored by direct visualization of EGFP fluorescence via microscopy and flow cytometry.

### Virus sources and preparation

A variety of HIV-1 isolates were used, including primary, laboratory-adapted, and reporter viruses, encompassing both CCR5-tropic and CCR5/CXCR4 dual-tropic strains. The following three viruses were obtained from the NIH AIDS Reagent Program: HIV ADA (ARP 416, CCR5-tropic), HIV 92/BR/014 (ARP 1753, Dual-tropic, syncytium-inducing), and HIV 92/BR/004 (ARP 1752, CCR5-tropic, non-syncytium-inducing).

EGFP-reporter viruses, specifically HIV (WT) IRES-EGFP (pBR43IeG nef+ R BaL env) and HIV (ΔNef) IRES-EGFP (pBR43IeG nef+ R BaL env, Nef deleted), were generated from plasmids generously provided by Dr. Thorsten Mempel and Dr. Shariq Usmani (39). These plasmids were transformed into Stbl2-competent *E. coli* for clonal selection and sequence confirmation. Confirmed plasmid clones were subsequently transfected into HEK 293T cells to produce viral stocks. The resulting virions were filtered and quantified for infectious titer using both p24 ELISA and TZM-bl assay.

### Transmission electron microscopy

Routine transmission electron microscopy (TEM) processing was done as described (116). In brief, primary human CD4^+^ T cells were negatively selected from freshly isolated PBMC and activated with 25 μL anti-CD3 and 25 μL anti-CD28 (Ultraleaf purified antibodies) in 10 mL RPMI + rhIL 2 (20 IU/mL) + IL15 . Cells were kept in a T25 flask, and media changes were done every 3–4 days for 22 days. These cells were split into two conditions: mock infected and HIV infected (ADA; TCID50/mL = 781,250 ). Infection was done by spinoculation at 2,900 rpm (1,965 × *g*) for 2 h prior to plating. 12 mL of media supplemented with IL2 and IL15 was used to plate CD4^+^ T cells (1 × 10^6^ cells/mL) onto 100 mm diameter cell culture-treated petri dishes that were previously coated with fibronectin. Following 5 days of infection, the cells were gently washed with phosphate-buffered saline and then fixed with 2.5 % glutaraldehyde in 0.1 M sodium cacodylate buffer (pH 7.4) at room temperature for 1 h. The cells were gently scraped off the 100 mm tissue culture-treated petri dish and pelleted by low-speed centrifugation (100 × *g* for 5 minutes). The pellet was fixed for 30 min with the same fixative before secondary fixation with 2 % osmium tetraoxide on ice for 1 h. The cells were then stained with 2% uranyl aqueous solution *en bloc* for 1 h at room temperature, dehydrated with a series of increasing ethanol gradients followed by propylene oxide treatment, and embedded in Embed 812 Resin mixture (Electron Microscopy Sciences). Blocks were cured for 48 h at 65°C and then trimmed into 70 nm ultrathin sections using a diamond knife on a Leica Ultracut 6 and transferred onto 200 mesh copper grids. Sections were counterstained with 1% uranyl acetate in 50% ethanol for 3 min at room temperature and in lead citrate for 3 min at room temperature, and then examined with a JEOL JSM 1400 transmission electron microscope equipped with two CCD cameras for digital image acquisition: Veleta 2K × 2K and Quemesa 11 megapixel (EMSIS, Germany) operated at 100 kV.

### Scanning electron microscopy

SEM was done on negatively selected primary human CD4^+^ T cells or cocultures with human myeloid cells grown in RPMI growth media and supplemented with 10% FBS, 20 IU/mL IL2, and antibiotics. Cells were plated on fibronectin-coated coverslips for 5 days. Adherent cells were fixed with 2.5 % glutaraldehyde and 1 % paraformaldehyde in a 0.12 M sodium cacodylate buffer for 20 min at room temperature, followed by a fixation for 1 hour in 1% osmium tetroxide (Electron Microscopy Sciences). The cells were dehydrated through a series of ethyl alcohol/deionized water solutions, followed by critical point drying and sputter coating with iridium. Imaging was done using a FEI Teneo LV SEM instrument (Thermo Fisher Scientific).

### Time-lapse confocal microscopy

Time-lapse confocal imaging was done in an incubated chamber. Image frames were taken at 1-s intervals with 25×, 40×, or 63× objectives. Videos were generated using a speed rate of 25 frames per second using Zeiss ZenBlue Software. When tracking organelle location using fluorescent dyes, 1 μM MitoTracker Deep Red FM (ThermoFisher, Catalog number: M22426) was used to detect mitochondria, and 1 μM LysoTracker Blue DND22 (ThermoFisher, Catalog number: L7525) was used to detect acidic organelles, including lysosomes and nuclei.

### Chemical inhibitions of actin regulators

Actin regulatory proteins were chemically inhibited in migrating primary CD4^+^ T cells to compare their morphologies to HIV-infected cells. CDC42 was inhibited by adding 10 μM ML 141 (Cayman Chemical, Catalog #18496). Rac proteins (Rac1, Rac1b, Rac2, and Rac3) were inhibited by adding 20 μM EHT 1864 (Cayman Chemical Company, Catalog #17258). Pak1/2 was inhibited by adding 50 μM IPA3 (Cayman Chemical Company, Catalog #14759 ). ARP2/3 was inhibited by adding 200 μM CK-666 (Cayman Chemical, Catalog #29038 ) to the media. P-REX1 was inhibited by adding 30 μM Compound # 5408-1697 (ChemDiv) (104).

### Nef transgenic mice

Primary tissues from CD4C/HIV Nef transgenic mice were used to isolate CD4+ T cells. These mice, also known as MutG, were previously bred on the C3H background (77). The cells express Nef constitutively and thus contain lower numbers of CD4+ T cells and show downregulation of cell surface CD4 protein. The spleens from the six mice were used, three Nef transgenic and three non-transgenic control mice. Spleens were shipped at 4°C and kept at 4°C until processing. The spleens were mechanically digested with sterile scissors, and cells were filtered through a 40 μm filter to get a single-cell suspension. 2% FBS in HBSS was used to wash the cells. Cells were spun down at 400 × *g* for 5 min and resuspended in 1 mL of Easysep buffer. Negative CD4^+^ T-cell isolation was then done using an EasySep Mouse CD4+ T-Cell Isolation Kit (StemCell Technologies #19852). Cells were then resuspended in 2 mL of RPMI + rhIL 2 + rhIL 15 (FBS + P/S/Q) and plated onto a microscope dish previously coated with fibronectin. All six spleen CD4^+^ T-cell samples were incubated at 37°C until imaging on the same day.

### Mass spectrometry—label-free quantitation

CD4^+^ T cells from 10 HIV-negative donors were isolated, activated, and infected with EGFP-containing reporter viruses. At 47 days post-infection, Mock, HIV WT EGFP, and HIV ΔNef EGFP CD4^+^ T cells were suspended at a concentration of 2 × 10^7^ cells/mL in complete media with IL2 and IL15 prior to cell sorting on a BD FACSAria II instrument. DAPI staining and mock-infected control cells were used to set gates for live and single cells. Cells positive for EGFP in HIV WT EGFP and HIV ΔNef EGFP cultures, along with bystander CD4^+^ T cells negative for EGFP, were all individually collected. Sorted populations were then washed three times in PBS, and cell pellets were stored at 80°C until mass spectrometry was initiated. Cell pellets were resuspended in 9 M urea, 50 mM Tris, pH 8.0. Proteomics analysis of HIV-infected and control T cells was done using label-free quantitation at the Weill Cornell Proteomics and Metabolomics Core. Proteins were precipitated using acetone before in-solution trypsin digestion was performed, followed by stagetip desalting and LCMS/MS. Each sample was analyzed using a data-independent acquisition (DIA ) method. The data were searched against a customized database containing Uniprot human protein sequences and nine target sequences, including HIV proteins and EGFP.

### Mass spectrometry—phosphosite analysis

A label-free proteomics workflow was used to detect the phosphorylation patterns in HIV-infected human T cells. Following sorting of live HIV-infected cells, the cells were washed three times with PBS and supernatant removed prior to pelleting and frozen at −80°C. Following sorting from eight separate donors (Table S3A), the cell pellets were lysed with the freshly prepared buffer (9 M urea with 5 mM NaF, 1 mM Na_3_VO_4_, 50 mM Tris at pH 8.0, and phosphatase/protease inhibitors) and pooled by condition due to protein yield. Three samples were sent to the core facility for analysis (HIV WT + Mock, HIVΔNef alone, and Mock alone). The low number of cells from the HIV WT condition required additional protein input for phosphosite analysis. This first sample thus contained HIV WT + Mock protein. Proteins were precipitated with acetone, and in-solution tryptic digestion was performed, followed by phosphopeptide enrichment. The phosphopeptides were subject to LC-MS/MS for analysis. MS data were searched against the UniProt human protein database, with a parameter setting phosphorylation as a dynamic modification. In total, 11,295 phosphosites were identified at a FDR of 1%. Identified phosphorylated peptides are shown in Table S3.

### Lymph node biopsies and human subjects IRB

Three lymph node tissues (cervical and inguinal) were analyzed from HIV-positive donors recruited at Centro de Investigación en Enfermedades Infecciosas (CIENI), Instituto Nacional de Enfermedades Respiratorias (INER) in Mexico City, Mexico. The lymph nodes were previously formalin-fixed and embedded in paraffin before sectioning and evaluation for spatial gene expression. The tissues were obtained in accordance with the Declaration of Helsinki after obtaining written informed consent of participants and as part of protocol B0316, which was reviewed and approved by the Research Committee and the Ethics in Research Committee of the National Institute of Respiratory Diseases “Ismael Cosío Villegas,” Mexico City.

### Spatial transcriptomics

#### FFPE tissues

The 10× Visium spatial transcriptomics protocol was followed for the procedure. All steps were performed in-house, with the exception of sequencing, which was performed by the Genomics and Epigenomics Core Facility of the WCM Core Laboratories Center. All PCR steps (with the exception of qPCR) were performed with the BioRad C100 Touch thermal cycler. Tissues were sectioned at 7 microns by microtome, and sections were placed on the gene expression slide provided with the 10× Visium kit. Tissues were deparaffinized according to protocol and stained with hematoxylin (MilliporeSigma, MHS16), bluing buffer (Ricca, 6697), and Eosin (Millipore Sigma, HT110116). Imaging was performed on a Keyence BZX810 following H& E staining. Tissue decrosslinking followed by probe hybridization, ligation, release, and extension was performed according to the 10× Visium protocol. Cycle determination qPCR was completed on a QuantStudio 7 Flex qPCR system (Applied Biosystems, 4485701). cDNA amplification was performed according to the protocol, with cDNA cleanup accomplished using SPRIselect beads (Beckman Coulter, B22318). Quality control was completed using an Agilent 2100 Bioanalyzer and high-sensitivity DNA chips (Agilent Technologies, 50674626 ). Completed libraries were sequenced at the Genomics and Epigenomics Core Facility of the WCM Core Laboratories Center.

### Sequencing

Visium Spatial Gene Expression libraries were generated from unique samples and individually tagged with unique identifiers. These libraries were pooled, and sequencing depth/spot was calculated based on the estimated coverage area of the tissue within each of the four grids. Paired-end, dual-indexed sequencing of four million total reads was done on an Illumina NextSeq500 platform using a 150-cycle kit. FASTQ files were generated with the Space Ranger (V1.0.0) package from 10× Genomics.

### Bioinformatics analysis of spatial transcriptomics data

The spatial transcriptomics data set, comprising 12 Visium sections from human lymph nodes (four sections from two viremic PLWH and four sections from one virally suppressed PLWH), underwent a comprehensive bioinformatics analysis to identify spatial gene expression patterns and cell-type specific transcriptional changes.

#### Data preprocessing and integration

Initial alignment and processing of the raw spatial transcriptomics data were performed using SpaceRanger (v2.1, 10× Genomics) to generate feature-barcode matrices and aligned H&E images. Subsequently, these processed data were imported into R (version 4.3.1) and ingested using the Seurat package (v5.0.1).

To integrate the 12 individual Visium samples, they were merged into a single Seurat object. Standard preprocessing steps were then applied:

- Normalization: gene expression counts were normalized using NormalizeData() (default LogNormalize method, scaling factor of 10,000).
- Feature selection: highly variable features were identified across the data set using FindVariableFeatures() (default vst method).
- Scaling: the data were scaled using ScaleData() to mitigate the influence of high-expression genes and prepare for dimensionality reduction.
- Dimensionality reduction: principal component analysis (PCA) was performed using RunPCA() on the scaled data, employing the identified highly variable features, to reduce dimensionality and extract the most significant sources of variation (default parameters were used for all these steps unless otherwise specified).

#### Cell type annotation

To accurately identify and annotate cell types within the Visium spatial spots, a reference-based transfer learning approach was utilized. A publicly available single-cell RNA sequencing (scRNA-seq) reference data set (117 ) was obtained. This reference data set, initially in H5AD format, was converted to the Seurat-compatible H5Seurat format using the Convert function. The pre-defined cell types from the reference scRNA-seq data set were then mapped onto the Visium spatial spots using Seurat’s transfer learning functionalities. Specifically, FindTransferAnchors() was used to identify anchors between the Visium data set and the reference, followed by TransferData() to project the cell type labels. This process utilized the first 30 principal components from the integrated data set for optimal accuracy in cell type prediction. Each Visium spot was assigned a “predicted.id” corresponding to a specific cell type.

#### Differential expression analysis (DEA)

To focus on CD4^+^ T-cell-specific changes, Visium spots annotated with CD4^+^ T-cell identities were isolated for downstream analysis. This included all spots predicted as T_CD4+_naive, T_CD4+_TfH, T_CD4+_TfH_GC, T_TfR, and T_Treg. Differential expression analysis was performed using FindMarkers() on these selected CD4^+^ T-cell-enriched spots. Spots were grouped according to the patient’s treatment status (ART-naïve viremic vs. virally suppressed on ART) to identify genes significantly differentially expressed between these conditions. A Wilcoxon Rank Sum test (test.use = “wilcox”) was employed, and genes with an adjusted *P*-value (Benjamini-Hochberg corrected) < 0.05 and a |log2 fold change| > 0.25 were considered significant.

#### Gene ontology enrichment analysis

The top differentially expressed genes identified from the DEA were subjected to gene ontology (GO) enrichment analysis using Metascape (76). This analysis was performed to identify over-represented biological processes, molecular functions, and cellular components associated with the transcriptional changes in CD4^+^ T cells during different stages of HIV infection.

## SUPPLEMENTAL MATERIAL

Legends mbio.03917-25-s0001.docx Supplemental file legends.

Supplemental Tables mbio.03917-25-s0002.xlsx Tables S1 to S3.

Video S1 mbio.03917-25-s0003.mp4 Uninfected human CD4+ T cell migration on 2-dimensional fibronectin-coated dishes.

Video S2 mbio.03917-25-s0004.mp4 HIV-infected cells exhibit rounded blebbing prior to apoptosis, while other infected cells form syncytia.

Video S3 mbio.03917-25-s0005.mp4 Rhino phenotype.

Video S4 mbio.03917-25-s0006.mp4 Polarized lamellipodial blebbing.

Video S5 mbio.03917-25-s0007.mp4 The effects of small molecule inhibitors of actin regulatory proteins on CD4+ T cell migration.

Video S6 mbio.03917-25-s0008.mp4 Abnormal morphologies are unique to HIV infected cells.

Video S7 mbio.03917-25-s0009.mp4 Nef contributes to actin disruption in primary CD4+ T cells.

Video S8 mbio.03917-25-s0010.mp4 Time-lapse microscopy showing the effects of a small molecule inhibitor of P-REX1 on CD4+ T cell migration.

ASM does not own the copyrights to Supplemental Material that may be linked to, or accessed through, an article. The authors have granted ASM a non-exclusive, world-wide license to publish the Supplemental Material files. Please contact the corresponding author directly for reuse.
