# Supplementary material for: HIV Nef-mediated WAVE2-ARP2/3 inhibition underlies CD4+ T-cell lamellipodial abnormalities and immune dysfunction
Source: mBio. 2026 Mar 30;17(5):e03917-25. doi: 10.1128/mbio.03917-25 (PMC13170354; doi:10.1128/mbio.03917-25)
Supplement: Legends — Supplemental file legends. [file mbio.03917-25-s0001.docx]

**Supplementary information:**

**Supplementary Table 1A – Clinical parameters of FFPE lymph node donors used for spatial transcriptomic analyses.** This table provides a summary of the clinical characteristics and treatment status (ART-naïve viremic vs. virally suppressed on ART) for the HIV-positive donors from whom FFPE (Formalin-Fixed Paraffin-Embedded) lymph node tissues were obtained for spatial transcriptomic profiling.

**Supplementary Table 1B – Top 20 up and down-regulated genes within spots containing CD4+ T cell clusters in FFPE lymph node spatial transcriptomic analysis.** This table presents the top 20 most significantly differentially expressed genes (DEGs), categorized as either upregulated or downregulated, within the spatially defined CD4+ T cell clusters identified in lymph node tissues from viremic PLWH compared to virally suppressed PLWH. It highlights key transcriptional changes associated with active HIV infection, including the decreased expression of ARP2/3 complex genes (ARPC1B, ARPC2, ARPC4).

**Supplementary Table 1C – Full list of DEGS within spots containing CD4+ T cell clusters in FFPE lymph node spatial transcriptomic analysis.** This comprehensive table provides the complete dataset of all differentially expressed genes identified within CD4+ T cell clusters during the spatial transcriptomic analysis of lymph node tissues from viremic versus virally suppressed PLWH. This includes statistical measures such as fold change, p-values, and adjusted p-values for each gene.

**Supplementary Table 2A – Clinical parameters of HIV‑ donors of CD4^+^ T cells used for infections and mass spec analysis.** This table details the demographic and clinical characteristics of the HIV-negative healthy donors from whom primary CD4+ T cells were isolated, activated, infected (or mock-infected), and subsequently used for bulk proteomics via label-free quantitative mass spectrometry.

**Supplementary Table 2B – Raw mass spectrometry reads of viral and host proteins expressed in Mock and sorted HIV‑infected primary human CD4^+^ T cells.** This table contains the raw quantitative data from label-free mass spectrometry, identifying and quantifying both host and viral proteins (including HIV proteins and EGFP) detected in mock-infected, HIV WT EGFP-infected, and HIV ΔNef EGFP-infected primary human CD4+ T cells after cell sorting. This dataset was used to assess overall protein expression levels.

**Supplementary Table 2C: Mass spectrometry data for proteins uniquely detected in HIV WT EGFP-infected primary human CD4+ T cells.**This table presents the raw label-free quantitative mass spectrometry data for proteins whose presence was uniquely detected in primary human CD4+ T cells infected with HIV WT EGFP. Specifically, it lists proteins identified in sorted EGFP-positive cells from wild-type HIV infections that were *not detected* in either mock-infected CD4+ T cells or in cells infected with the HIV ΔNef EGFP virus. This dataset highlights host proteins that are exclusively associated with productive wild-type HIV infection and the presence of Nef, potentially indicating their specific involvement in Nef-mediated cellular processes.

**Supplementary Table 2D: Gene Ontology (GO) enrichment analysis of proteins uniquely detected in HIV WT EGFP-infected primary human CD4+ T cells.** This table provides the results of Gene Ontology (GO) enrichment analysis performed on the set of proteins uniquely detected in HIV WT EGFP-infected primary human CD4+ T cells (as identified in Supplementary Table 2C). The analysis (performed using Metascape as mentioned in the Bioinformatics Methods section) categorizes these proteins by their enriched biological processes, molecular functions, and cellular components.

**Supplementary Table 3A – Demographic parameters of HIV‑ donors of CD4^+^ T cells used for infections and phosphosite mass spec analysis.** This table provides the demographic and clinical information for the HIV-negative healthy donors whose primary CD4+ T cells were used for in vitro infection experiments followed by phosphosite mass spectrometry analysis to investigate post-translational modifications.

**Supplementary Table 3B – Raw mass spectrometry reads of phosphosites detected in Mock and sorted HIV‑infected primary human CD4^+^ T cells.** This extensive table presents the raw mass spectrometry data for all identified phosphosites in proteins from mock-infected, HIV WT EGFP-infected, and HIV ΔNef EGFP-infected primary human CD4+ T cells. It includes information on the specific protein, amino acid residue, and phosphorylation status across the experimental conditions.

**Supplementary Table 3C – Mass spectrometry reads of phosphosites detected only in HIV-WT expressing cells and not detected in Mock DNef-infected cells.** This focused table highlights phosphosites that were uniquely detected or significantly elevated in primary CD4+ T cells infected with HIV WT EGFP virus, but not in mock-infected or HIV ΔNef EGFP-infected cells. This subset is crucial for identifying phosphorylation events specifically mediated by the Nef protein during HIV infection.

**Supplementary Table 3D – Mass spectrometry reads of phosphosites in ARP2/3, NPF, and Lamellipodial proteins.** This table compiles the mass spectrometry data related to phosphosites on proteins involved in the ARP2/3 complex, Nucleation Promoting Factors (NPFs), and other lamellipodial actin regulators. It focuses on identifying post-translational modifications in these key cytoskeletal components under various infection conditions (mock, HIV WT, HIV ΔNef).

**Supplementary Video 1:** **Uninfected human CD4^+^ T cell migration on 2‑dimensional fibronectin coated dishes (time‑lapse, SEM, & TEM).** This video captures the normal polarized morphology and dynamic movement of a healthy primary human CD4+ T cell. Observe the continuous extension of a broad lamellipodium and the coordinated retraction of the uropod, illustrating efficient chemotaxis. (Speed rate: 25 frames per second, original frames taken at 1-second intervals).

**Supplementary Video 2: HIV infected cells exhibit rounded blebbing prior to apoptosis (time‑lapse & TEM), while other infected cells form syncytia.** This video illustrates the dynamic formation and retraction of membrane blebs on the surface of an HIV-infected CD4+ T cell. This phenotype is indicative of significant cortical actin disruption and, in some contexts, precedes apoptosis. The second part of this video demonstrates the fusion of multiple HIV-infected CD4+ T cells to form large, multinucleated syncytia. This cytopathic effect is observed with both R5 and R5/X4 tropic viruses and contributes to CD4+ T cell depletion. (Speed rate: 25 frames per second, original frames taken at 1-second intervals).

**Supplementary Video 3: Rhino phenotype (time‑lapse & TEM).** This video demonstrates another unique lamellipodial defect observed in HIV-infected CD4+ T cells, characterized by the formation of an elongated and pointed lamella/lamellipodium, termed the "Rhino" phenotype. This phenotype is also indicative of impaired ARP2/3 activity. (Speed rate: 25 frames per second, original frames taken at 1-second intervals).

**Supplementary Video 4: Polarized lamellipodial blebbing (time‑lapse & TEM).**  This video highlights a distinct morphological abnormality in HIV-infected CD4+ T cells, characterized by dynamic blebbing occurring specifically at the leading edge (lamellipodium) while maintaining overall cell polarity. This phenotype is strongly associated with ARP2/3 inhibition. (Speed rate: 25 frames per second, original frames taken at 1-second intervals).

**Supplementary Video 5: The effects of small molecule inhibitors of actin regulatory proteins on CD4^+^ T cell migration.** This video presents the morphological consequences of inhibiting CDC42 (ML-141), Rac proteins (EHT-1864), or Pak1/2 (IPA-3) in primary human CD4+ T cells. It serves to differentiate these effects from the specific lamellipodial abnormalities observed during HIV infection, most of which do not fully recapitulate the HIV-induced phenotypes. (Speed rate: 25 frames per second, original frames taken at 1-second intervals). This video also demonstrates that direct chemical inhibition of the ARP2/3 complex with CK-666 in uninfected CD4+ T cells strikingly induces both the polarized lamellipodial blebbing and the "Rhino" phenotypes. (Speed rate: 25 frames per second, original frames taken at 1-second intervals).

**Supplementary Video 6: Abnormal morphologies are unique to HIV infected cells (HIV WT EGFP^+^), (time‑lapse).** This video shows co-cultures of HIV WT EGFP-infected and bystander (uninfected) CD4+ T cells. It clearly illustrates that the "Rhino" and polarized lamellipodial blebbing phenotypes are exclusively observed in EGFP-positive (infected) cells, while adjacent EGFP-negative cells maintain normal migratory behaviors. (Speed rate: 25 frames per second, original frames taken at 1-second intervals).

**Supplementary Video 7: Nef Contributes to Actin Disruption in Primary CD4^+^ T cells.** This first half of the video compares the morphology of CD4+ T cells infected with a ΔNef EGFP reporter virus to those infected with wild-type virus. While the "Rhino" phenotype is largely absent in ΔNef-infected cells, some residual polarized lamellipodial blebbing still occurs, indicating that Nef is a major, but not sole, viral determinant of these actin abnormalities. (Speed rate: 25 frames per second, original frames taken at 1-second intervals). The second part of the video provides evidence that HIV Nef alone is sufficient to induce lamellipodial abnormalities. CD4^+^ T cells constitutively expressing Nef from CD4C/HIV Nef transgenic mice exhibit polarized blebbing, a phenotype highly similar to that seen in human T cells infected with wild-type HIV. (Speed rate: 25 frames per second, original frames taken at 1-second intervals). Non-transgenic control cells exhibited standard morphologies of non-infected CD4+ T cells (data not shown).

**Supplementary Video 8: Time-lapse microscopy showing the effects of a small molecule inhibitor of P-REX1 on CD4^+^ T cell migration.**This video illustrates the morphological and migratory changes in CD4+ T cells upon inhibition of P-REX1, a RAC-GEF identified as differentially phosphorylated in Nef-expressing cells. P-REX1 bridges mTOR and Rac2 to the WAVE2 complex. (Speed rate: 25 frames per second, original frames taken at 1-second intervals).
